# Partially Bio-Based and Biodegradable Poly(Propylene Terephthalate-Co-Adipate) Copolymers: Synthesis, Thermal Properties, and Enzymatic Degradation Behavior

**DOI:** 10.3390/polym16182588

**Published:** 2024-09-13

**Authors:** Ping Song, Mingjun Li, Haonan Wang, Yi Cheng, Zhiyong Wei

**Affiliations:** 1 School of Materials Science and Engineering, North University of China, Taiyuan 030051, China; songping@nuc.edu.cn; 2 Department of Polymer Science and Engineering, School of Chemical Engineering, Dalian University of Technology, Dalian 116024, China

**Keywords:** bio-based copolymers, thermal degradation kinetics, crystallization kinetics, enzymatic degradation

## Abstract

A series of partially bio-based and biodegradable poly(propylene terephthalate-co-adipate) (PPTA) random copolymers with different components were prepared by the melt polycondensation of petro-based adipic acid and terephthalic acid with bio-based 1,3-propanediol. The microstructure, crystallization behavior, thermal properties, and enzymatic degradation properties were further investigated. The thermal decomposition kinetics was deeply analyzed using Friedman’s method, with the thermal degradation activation energy ranging from 297.8 to 302.1 kJ/mol. The crystallinity and wettability of the copolymers decreased with the increase in the content of the third unit, but they were lower than those of the homopolymer. The thermal degradation activation energy *E*, carbon residue, and reaction level *n* all showed a decreasing trend. Meanwhile, the initial thermal decomposition temperature (*T_d_*) was higher than 350 °C, which can meet the requirements for processing and use. The PPTA copolymer material still showed excellent thermal stability. Adding PA units could regulate the crystallinity, wettability, and degradation rate of PPTA copolymers. The composition of PPTA copolymers in different degradation cycles was characterized by ^1^H NMR analysis. Further, the copolymers’ surface morphology during the process of enzymatic degradation also was observed by scanning electron microscopy (SEM). The copolymers’ enzymatic degradation accorded with the surface degradation mechanism. The copolymers showed significant degradation behavior within 30 days, and the rate increased with increasing PA content when the PA content exceeded 45.36%.

## 1. Introduction

The billions upon billions of items of plastic waste choking our oceans, lakes, and rivers, and piling up on land, are more than just unsightly—they are harmful to plants and wildlife. Plastic pollution is a very real issue, and single-use plastics, though small, have a large impact. To address plastic pollution, some biodegradable aliphatic–aromatic copolyesters, such as poly(butylene adipate-co-terephthalate) (PBAT) [1,2] and poly(butylene succinate-co-terephthalate) (PBST) [3,4], have already been successfully commercialized. Simultaneously, many studies on copolymerization modification based on different aromatic polyesters, which introduce aliphatic units, have also been reported. In a study by Chen et al. [5], a ternary copolymerization approach utilizing dimethyl terephthalate (DMT), 1,4-butanediol (BDO), and adipic acid (AA) was employed. It was observed that when the feed molar ratio of DMT to AA was 1:1, the resulting copolyester exhibited the lowest crystallinity (20%) and highest biodegradability under composting conditions. Berti et al. [6] used BDO, 1,12-dodecanedioic acid (DA), and terephthalic acid (PTA) as raw materials to prepare a series of random aliphatic–aromatic copolyesters by adjusting the molar ratio of DA to PTA. When the molar content of PTA is 70%, the melting point of the copolyester reaches as high as 186 °C, significantly enhancing heat resistance, but biodegradability deteriorates.

With the increasing consumption of fossil resources, the concept of sustainable development has emerged in response to environmental pollution and energy depletion. Therefore, researchers are now focused on vigorously developing and utilizing renewable resources [7,8,9,10,11]. 1,3-Propanediol, regarded as a green raw material, can be obtained from renewable glycerol through microbial fermentation and used as a monomer with PTA to synthesize polypropylene terephthalate (PPT) through melt polycondensation [12,13,14]. This semi-crystalline thermoplastic polyester is extensively used in the production of textile fibers and films because it possesses the excellent properties of both polyamide and polyester, such as good elasticity and dimensional stability [15].

Despite its relatively outstanding performance, PPT poses a significant environmental threat due to its non-biodegradability. As PPT production capacity continues to expand, improper recycling practices may lead to its persistent presence in the environment, causing severe white pollution. Aliphatic–aromatic copolyesters are biodegradable materials with better mechanical and thermal properties than aliphatic polyesters [16,17,18]. The introduction of AA as an aliphatic copolymer unit in PPT polyesters has the potential to render PPT biodegradable, enabling the development of a novel bio-based material that holds both environmental and economic value.

In this work, a series of partially bio-based and biodegradable poly(propylene terephthalate-co-adipate) (PPTA) copolymers with different compositions were synthesized using bio-based 1,3-propanediol. The microstructure, molecular weight and distribution, crystallization behavior, thermal properties, and surface hydrophilicity were analyzed and studied. Furthermore, the enzymatic degradation behavior of the copolymers was investigated. This work offers a feasible approach for developing biodegradable general-purpose plastics with a high bio-based content. The developed PPTA copolymers hold promise as a potential replacement for unsustainable petroleum-based polymers in future markets.

## 2. Experimental

### 2.1. Materials

Purified terephthalic acid (PTA) and adipic acid (AA) were kindly provided from Kanghui New Material Technology Co., Ltd., Yingkou, China. Bio-based 1,3-propanediol (PDO, >99%) was purchased from Guangdong Qingda Zhixing Biotechnology Co., Ltd., Dongguan, China. Phenol, chemically pure, was purchased from Aladdin Reagent Co., Ltd., Shanghai, China. Tetrabutyl titanate (TBT), analytically pure, was purchased from Aldrich Co., Ltd., St. Louis, MI, USA. 1,1,2,2-Tetrachloroethane, analytically pure, was purchased from Maclean’s Reagent Co., Ltd., Allen Park, MI, USA. Trifluoroacetic acid, analytically pure, was purchased from Aladdin Reagent Co., Ltd. Deuterated chloroform, of analytical purity, was purchased from Aladdin Reagent Co., Ltd. Lipase PS (≥23,000 U/g) was purchased from Aladdin Reagent Co., Ltd. (the optimal temperature is 50 °C, and the optimal pH is 7.0).

### 2.2. Preparation of Poly(Propylene Terephthalate-Co-Adipate) Copolymers

**Figure 1:**
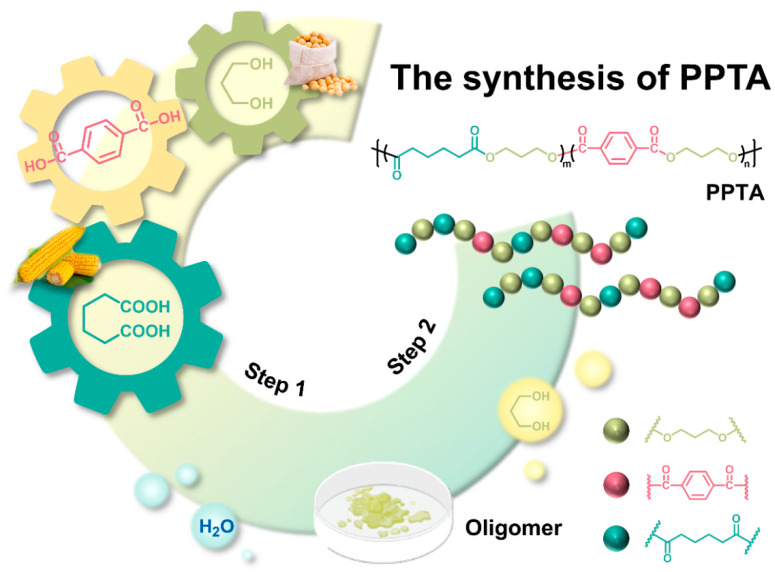
Synthetic routine of PPTA copolymers.

Poly(propylene terephthalate-co-adipate) copolymers with different compositions were prepared by controlling different feeding ratios (PTA:AA) using bio-based 1,3-propanediol (PDO) as the diol and terephthalic acid (PTA) and adipic acid (AA) as the diacid, as shown in Figure 1. Firstly, weighed PTA, 1,3-PDO, AA (molar ratio of alcohol to acid = 1.8:1), and a catalyst (TBT, based on 0.1 mol% of the total acid content) were added to a 100 mL four-neck flask. Subsequently, the four-neck flask, mechanical stirring, thermometer, condenser tube, distillation column, and nitrogen device were fixed in turn. Nitrogen protection and mechanical stirring were turned on, and the heating jacket temperature was set to 100 °C. The temperature at the top of the column was controlled at about 100 °C with the first drop of water generation time as the esterification start time during the temperature rise. The temperature was raised to the condensation temperature during the condensation stage, and the vacuum was less than 100 Pa. Finally, the reaction was stopped when the torque reached the maximum. The synthetic feeding ratios, esterification temperature, esterification time, polycondensation temperature, and polycondensation time are summarized in Table 1. The samples were named according to the molar percentage of adipic acid (AA) in the total acid content as PPTA-20, PPTA-40, PPTA-60, PPTA-80, and PPA.

### 2.3. Enzymatic Degradation Experiment

The samples were pressed and cut into 1 cm × 1 cm × 0.5 cm pieces at 250 °C. An enzyme solution of 0.5 mg/mL was prepared using PBS phosphate buffer (pH = 7.2–7.4) as the solvent [19]. The pieces were put into a centrifuge tube with the enzyme solution, and then the centrifuge tube was put into a thermostatic culture shaker with the temperature set to 50 °C. All samples were changed every 8 days, and a set of samples were washed three times with distilled water every 4 days and dried under vacuum at 50 °C for 48 h. The mass loss rate of the samples was calculated by the following equation:

(1) $$Mass\;loss\left(\%\right)=\frac{M_{1}-M_{2}}{M_{1}}\times 100\%$$

where *M*_1_ is the mass of the sample before degradation and *M*_2_ is the mass of the sample after degradation at different periods. The average value and error were calculated.

### 2.4. Characterization

#### 2.4.1. Nuclear Magnetic Resonance (NMR)

Nuclear magnetic resonance (NMR) spectra were measured using a Vaian DLG400 NMR spectrometer. A mixture of trifluoroacetic acid (CF_3_COOH) and deuterated chloroform (CDCl_3_) was used as the solvent, which was 0.1 g/mL with TMS as the internal standard.

#### 2.4.2. Intrinsic Viscosity [*η*]

The intrinsic viscosity [*η*] of the polymer was characterized using an Ubbelohde viscometer with a phenol/1,1,2,2-tetrachloroethane (60:40 *w*/*w*) as the solvent. A sample weighing about 0.2 g was used to configure a solution with a concentration of 10 g/L in a constant temperature water bath at 25 °C.

#### 2.4.3. Gel Permeation Chromatography (GPC)

The molecular weight and distribution were characterized using ambient viscous gel permeation chromatography (GPC) by Waters, Milford, MA, USA. Tetrahydrofuran (THF) was employed as a flow term with a flow rate of 1.0 mL/min at 35 °C.

#### 2.4.4. Wide Angle X-ray Diffraction Patterns (WAXD)

The X-ray diffraction patterns were analyzed using a Smart Lab 9 KW intelligent X-ray diffractometer under Cu target radiation (voltage = 240 kV, current = 50 mA, *λ* = 0.02 nm, 2*θ* is 10°~35°). The samples were pressed using a press before the test, isothermally crystallized at 150 °C for 12 h, and then annealed.

#### 2.4.5. Differential Scanning Calorimetry (DSC)

Non-isothermal crystallization: Measurements were performed using a TA Q25 differential scanning calorimeter from the USA under a nitrogen atmosphere with a range of −50 to 260 °C. About 5 mg of the dried sample was weighed and heated to 260 °C at 10 °C/min under a nitrogen atmosphere at 50 mL/min and maintained in an isothermal state for 3 min to eliminate the thermal history. Then, the temperature was lowered to −50 °C at 10 °C/min and finally heated to 260 °C at 10 °C/min.

Isothermal crystallization: About 5 mg of the dried sample was weighed and ramped up to 260 °C at a rate of 10 °C/min under a nitrogen atmosphere of 50 mL/min and maintained in an isothermal state for 3 min to eliminate thermal history. Then, it was cooled down to isothermal crystallization temperature *T*_c_ at a 100 °C/min cooling rate and stayed so for 1 h.

#### 2.4.6. Thermogravimetric Analysis (TGA)

The thermal decomposition properties of the polymers were characterized using a TA Q500 thermogravimetric analyzer. About 5 mg of the dried sample was weighed into the crucible and heated up to 800 °C at a heating rate of 10 °C/min under a nitrogen atmosphere of 50 mL/min.

#### 2.4.7. Water Contact Angles (WCA)

The water contact angles were measured using a contact angle measuring instrument. The samples were melted and pressed at 250 °C and then placed on the carrier table of the contact angle measuring instrument. Deionized water was slowly squeezed out on the samples, after which the contact angle values were photographed and recorded. Three experiments were conducted for each group, and the error and average values were calculated.

#### 2.4.8. Scanning Electron Microscopy (SEM)

The material after enzymatic degradation was cut into sample pieces of about 2 mm × 2 mm, and the sample pieces were fixed to the conductive adhesive and sprayed with gold. The surface morphology of the samples was observed using a QUANTA 450 tungsten scanning electron microscope (SEM) produced by FEI, San Jose, CA, USA.

## 3. Results and Discussion

### 3.1. Synthesis and Structure Characterization of the PPTA Copolymers

A series of bio-based PPTA copolymers were synthesized by the melt polycondensation method, and their microstructures were characterized by ^1^H NMR characterization. The ^1^H NMR spectra of the PPTA-40 copolymer were attributed as Figure S1. There are TPT, APA, and TPA: three possible sequence structures. The chemical shifts of trifluoroacetic acid and deuterated chloroform appeared at 11.07 ppm and 7.26 ppm, respectively. The hydrogen on the benzene ring attached to the ester group appeared at δ = 8.20 ppm. When the sequence structure was TPT, triple and quintuple peaks appeared at δ = 4.66 ppm and δ = 2.25 ppm, respectively, for propylidene (-CH_2_CH_2_CH_2_-O-) linked to oxygen. When the sequence structure was APA, triple and quintuple peaks appeared at δ = 2.49 ppm and δ = 1.71 ppm, respectively, for butylidene linked to the ester group. Additionally, the propylidene linked to oxygen, with triple and quintuple peaks at δ = 4.28 ppm and δ = 2.08 ppm, respectively. In addition, triple and quintuple peaks at δ = 4.55 ppm, δ = 2.25 ppm/δ = 2.08 ppm, and δ = 4.39 ppm were recorded for the oxygen-linked propylidenes when the sequence structure was TPA. The sequence structures and the degree of randomness (*R*) were determined by the following equations:

(2) $$X_{PA}=\frac{I_{B}}{I_{A}+I_{B}}$$

(3) $$X_{PT}=\frac{I_{A}}{I_{A}+I_{B}}$$

(4) $$L_{PT}=\frac{I_{a}+I_{b}}{I_{b}}$$

(5) $$L_{PA}=\frac{I_{b}+I_{c}}{I_{c}}$$

(6) $$R=\frac{1}{L_{PT}}+\frac{1}{L_{PA}}$$

where *X_PA_* represents the molar content of adipate; *X_PT_* is the molar content of terephthalate units; *L_PT_* and *L_PA_* are the average sequence lengths of PT and PA; and *I_a_*, *I_b_*, and *I_c_* are the integrals of the corresponding peaks.

**Figure 2:**
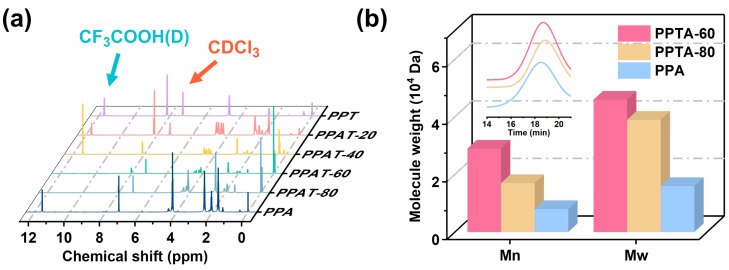
(**a**) ^1^H NMR and (**b**) GPC spectra of PPTA copolymers.

The results of intrinsic viscosity, feeding ratio, copolymer composition, molecular weight, and molecular weight distribution are summarized in Table 2. The calculated feeding ratios did not differ significantly from the composition measured by ^1^H NMR, proving the successful synthesis of the target product. As we know, the type of copolymer can be evaluated by the size of the randomness, which is 1 for random copolymers, 2 for alternating copolymers, and 0 for block copolymers. The calculated polymer randomness was close to 1, indicating that the synthesized PPTA copolymers were random. The GPC spectra are shown in Figure 2b. The molecular weights and viscosities of different compositions were investigated, and it was found that when the PA content was lower than 57.5%, the polymer could not be dissolved in tetrahydrofuran solution, so GPC characterization could not be performed.

### 3.2. Non-Isothermal and Isothermal Crystallization

**Figure 3:**
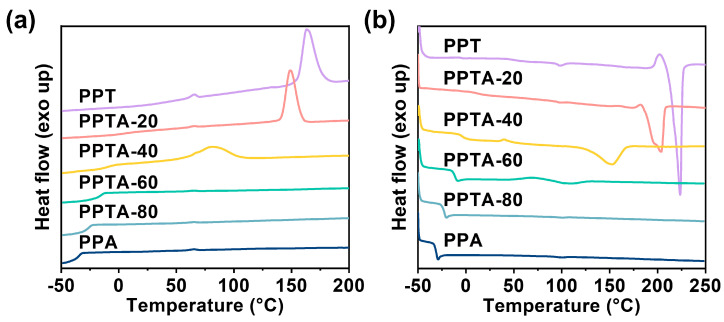
(**a**) Cooling curves and (**b**) the second heating curves for PPTA copolymers.

The thermal properties were investigated by DSC. Figure 3a,b show the DSC cooling curves and the second heating curves, respectively. As shown in Figure 3, the melting point (*T_m_*) of the copolymers decreased and the crystallization peak also moved to a low temperature when the PA content increased and the half-peak width of the crystallization peak was gradually becoming larger. The glass transition temperature (*T_g_*) also decreased with the increase in PA content. The appearance of a cold crystallization peak depends on the cooling rate and the crystallization ability of the material. In material which is difficult to crystallize, it is easier to observe the cold crystallization peak during the second heating curve. The PPTA copolymer began to show a cold crystallization peak and the melting peak gradually disappeared when the PA content was 57.5%. Furthermore, the crystallization ability and the crystallinity decreased with the increase in PA content, which is due to the insertion of PA chain segments destroying the regularity of the main chain, thereby making the crystallinity and crystallization rate decrease [20]. Semi-crystalline polymers usually contain both crystalline and amorphous regions. In order to describe this state quantitatively, the concept of crystallinity is proposed. Crystallinity can usually be characterized by X-ray diffraction [21,22]. The corresponding non-isothermal data and crystallinity (*X*_c_) are summarized in Table S1. PPTA-80 behaves as an amorphous polymer at this heating and cooling rate. Additionally, the crystallinity of PPTA copolymers decreased with the increase in the third unit content.

The crystallization behavior of polymers has always been the focus of attention, and among the various methods to react to the crystallization behavior of polymers, crystallization kinetics [23,24,25,26] has always been of interest. The effect of the composition of PPTA on its crystallization behavior was further investigated using isothermal crystallization kinetics. The copolymer was amorphous, and the crystallization behavior could not be studied when the PA content was greater than 45.36%. Therefore, PPTA-20 and PPTA-40 were selected to investigate the crystallization kinetics in the temperature range of 162–168 °C and 130–136 °C, respectively. The Avrami equation [27,28] was applied to analyze the isothermal crystallization kinetics.

(7) $$1-X_{t}=e^{{-kt}^{n}}$$

(8) $$t_{1/2}={\left(\frac{ln2}{k}\right)}^{\frac{1}{n}}$$

where *X_t_* is the relative degree of crystallinity, *k* is the overall kinetic constant, *t* is the crystallization time, *t*_1/2_ is the half crystallization time, and *n* represents the Avrami exponent, which is associated with the nucleation mechanism and growth dimension.

**Figure 4:**
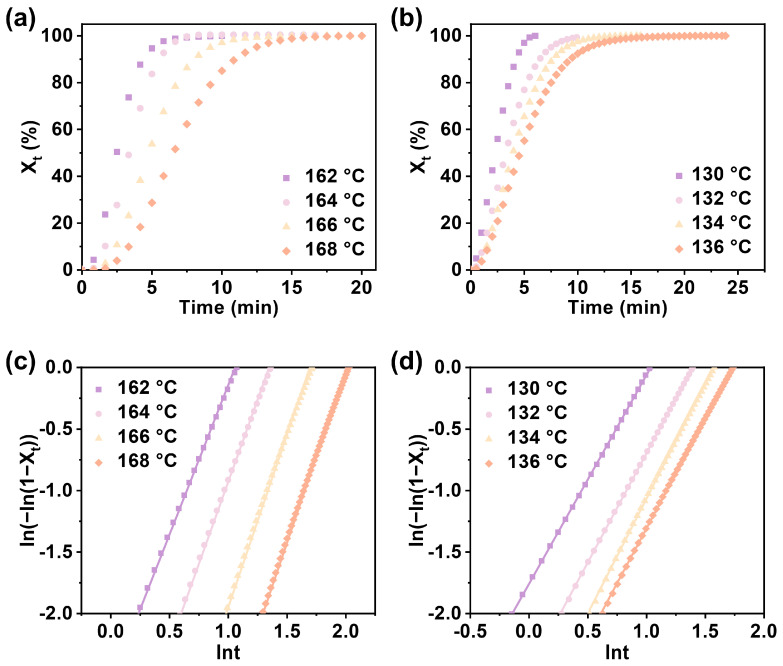
The relative crystallinity of (**a**) PPTA-20 and (**b**) PPTA-40 changes with time at different isothermal crystallization temperatures. Avrami analysis by plotting *ln*(−*ln*(l − *X*_t_)) vs. ln*t* of (**c**) PPTA-20 and (**d**) PPTA-40 at various *T*_c_ values.

It can be seen from Figure 4a,b that the relative crystallinity of all samples at different isothermal crystallization temperatures shows an S-shaped curve with time. The higher the isothermal crystallization temperature, the longer the crystallization time. The half-crystallization time of the same sample increases with the increase in the crystallization temperature, which conforms to the characteristics of crystallization kinetics. As shown in Figure 4c,d, the Avrami curves of all samples at different isothermal crystallization temperatures are linear, and the Avrami plots of the same sample at different isothermal crystallization temperatures are parallel. The crystallization kinetic data for copolymers of different compositions are summarized in Table S2. As shown in Table S2, the crystallization rate decreases and the crystallization activation energy increases significantly when the PA content increases. This is due to the addition of a third monomer, adipic acid, which disrupts the regularity of the polymer molecular chain structure and thus reduces the crystallization ability. And the higher the crystallization activation energy, the greater the obstacles to be overcome for crystallization, and the less likely the molecules will crystallize, which is consistent with the conclusions we obtained.

### 3.3. Crystal Structure of PPTA Copolymers

The crystal structure of PPTA copolymers was investigated by the WAXD technique. The samples were pressed using a press before the test, isothermally crystallized at 150 °C for 12 h, and then annealed. The WAXD patterns are depicted in Figure S2. All the annealed polymer materials exhibit diffraction peaks with different intensities. As shown in Figure S2, the diffraction peaks of the polymers were similar to PPT at lower PA content, which indicates that the crystalline shape of the polymers did not change. The intensity of the diffraction peaks gradually weakened with increasing PA content. It means that the crystallinity decreased with the increase in PA content, which is consistent with the results obtained by DSC. Therefore, the crystalline properties of copolymers can be effectively regulated by changing the PA content.

### 3.4. Thermal Stability and Thermal Degradation Kinetics of PPTA Copolymers

Thermal stability is an essential factor affecting the performance of polymers. Thermal degradation kinetics is a crucial index to evaluate the thermal properties of polymers. In the thermal degradation process, the thermodynamic equation can be expressed by the Arrhenius equation:

(9) $$\frac{d\alpha }{dt}=Z{\left(1-\alpha \right)}^{n}e^{-E/(RT)}$$

where *α* is the reaction conversion rate and *dα*/*dt* represents the thermal degradation rate, i.e., the ratio of weight loss at moment *t* to the total mass loss at the end of the reaction. *Z* is the reaction rate constant. *E* is the behavior of the thermal degradation activation energy. *R* is the gas constant with a value of 8.314 J·mol^−1^·K^−1^, and *T* represents the absolute temperature in *K*.

There are many methods to calculate the kinetic parameters of the thermal degradation of polymers, but dynamic thermal analysis is the main method. Friedman’s method [29,30] is a common analytical method to deal with TG and DTG curves at the same heating rate, so as to obtain the kinetic parameters of thermal degradation, whose equation is shown as follows. Plotting *ln*(*dα*/*dt*) and *ln*(1 − *α*) against 1/*T*, respectively, two straight lines can be obtained, and the magnitudes of *E* and *n* can be obtained by calculating and analyzing the slope and intercept of the lines.

(10) $$\ln\left(Z\right)=\ln\left(\frac{d\alpha }{dt}\right)-nln\left(1-\alpha \right)+\frac{E}{RT}$$

**Figure 5:**
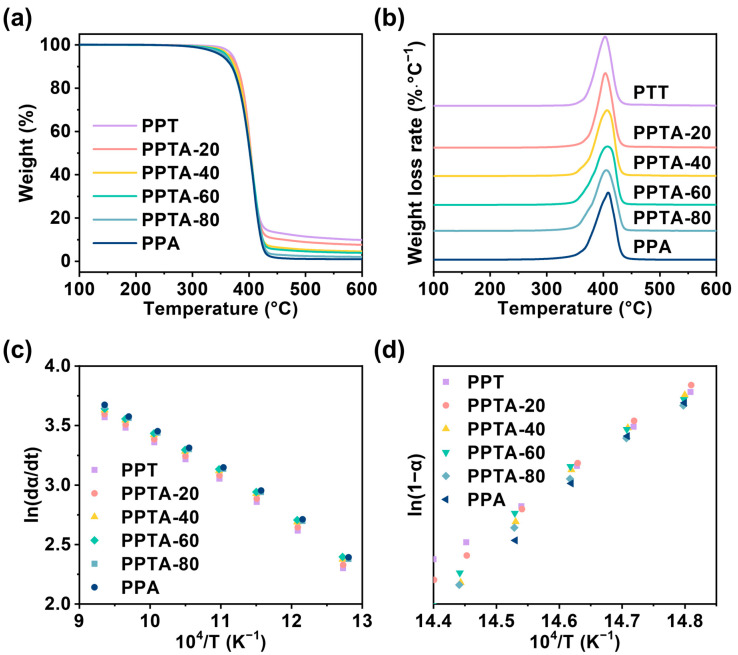
(**a**) Thermogravimetric analysis (TGA), (**b**) derivative thermogravimetry (DTG), (**c**) ln(dα/dt) vs. 10^4^/T curves, and (**d**) *ln*(1 − α) vs. 10^4^/T curves of PPTA with different compositions.

The thermal degradation properties of different components of PPTA copolymers were investigated separately. Thermogravimetric analysis (TGA) mass loss curves and derivative thermogravimetry (DTG) thermograms are shown in Figure 5. The kinetics of the thermal decomposition of copolymers was analyzed by the Friedman method. To facilitate the calculation, *ln*(*dα*/*dt*) and *ln*(1 − *α*) were plotted against 10^4^/*T*, respectively. By calculating and analyzing the slope and intercept of the line, the magnitude of *E* and *n* can be obtained. The corresponding thermal degradation kinetics data for PPTA with different compositions are summarized in Table S3. The data points were all linear, which indicated that the analysis of thermal degradation kinetics by Friedman’s method was feasible. In detail, *T_d_* was the thermal decomposition temperature and *T_dm_* was the fastest thermal degradation temperature. In general, the higher the activation energy of thermal degradation, the stronger the thermal stability, and the smaller the number of reaction levels *n*. According to Table S3, the activation energy of thermal degradation ranged from 297.8 to 302.1 kJ/mol. The thermal degradation activation energy *E*, carbon residue, and reaction level *n* of the copolymers kept decreasing with the increase in PA content in the copolymer, which means that the addition of a PA unit weakened the thermal stability performance of the material. However, the initial thermal decomposition temperature (*T_d_*) was higher than 350 °C, which can meet the requirements for processing and using this material. The PPTA copolymer material still showed excellent thermal stability.

### 3.5. Enzymatic Degradation Test

**Figure 6:**
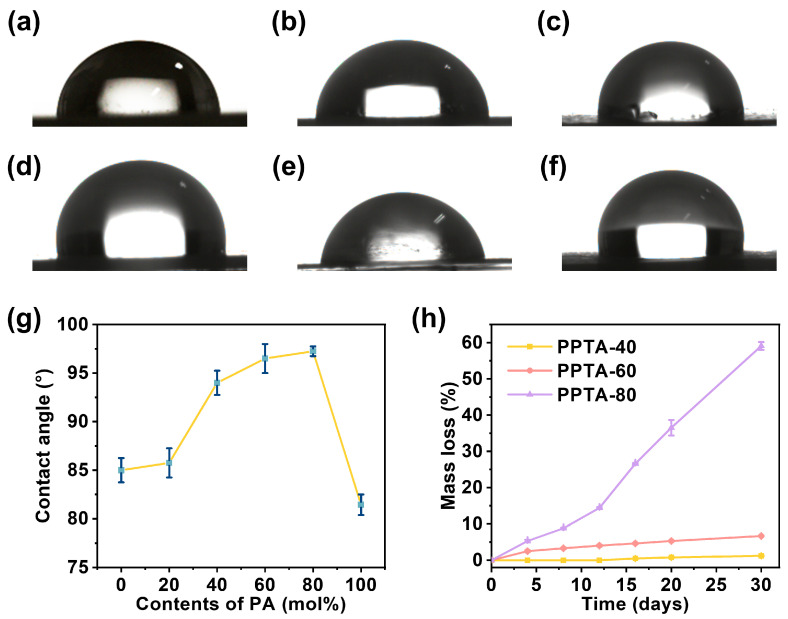
Contact angle photographs of (**a**) PPT, (**b**) PPTA-20, (**c**) PPTA-40, (**d**) PPTA-60, (**e**) PPTA-80, and (**f**) PPA. (**g**) Diagram of contact angle changing with PA content. (**h**) Curves of weightlessness of PPTA with different compositions over time.

The contact angle of liquid on the surface of a solid material is an important parameter to measure the surface-wetting performance of the material [31,32,33]. The water contact angles of different compositions of PPTA are presented in Figure 6. The material appears hydrophobic when the contact angle is greater than 90°, and it appears hydrophilic when the contact angle is less than 90°. The material is more wettable when the contact angle is smaller. PPT and PPA homopolymers had better wettability compared with PPTA copolymers. In addition, copolymers with PA content between 45.4% and 79.8% exhibit hydrophobic materials. Within a certain range, as the proportion of PA in the polymer increases, the water contact angle gradually increases. This is due to the addition of PA components destroying the regularity of the polymer, thus weakening the hydrophilicity of the copolymers. It can be seen that the hydrophilicity of the material can be adjusted by changing the PA content, which is important for the practicality of the material.

Biodegradability is one of the important properties of degradable polyester materials, and the degradation cycle is an important factor affecting the range of material applications [34]. Therefore, we investigated the enzymatic degradation behavior of PPTA copolymers. According to the curves of weight loss with time in Figure 6h, the copolymers all showed significant degradation behavior within 30 days, and the degradation rate was accelerated with the increase in PA content when the PA content was higher than 45.36%. The weight loss rate of PPTA-80 was as high as 59.1% after 30 days of degradation.

**Figure 7:**
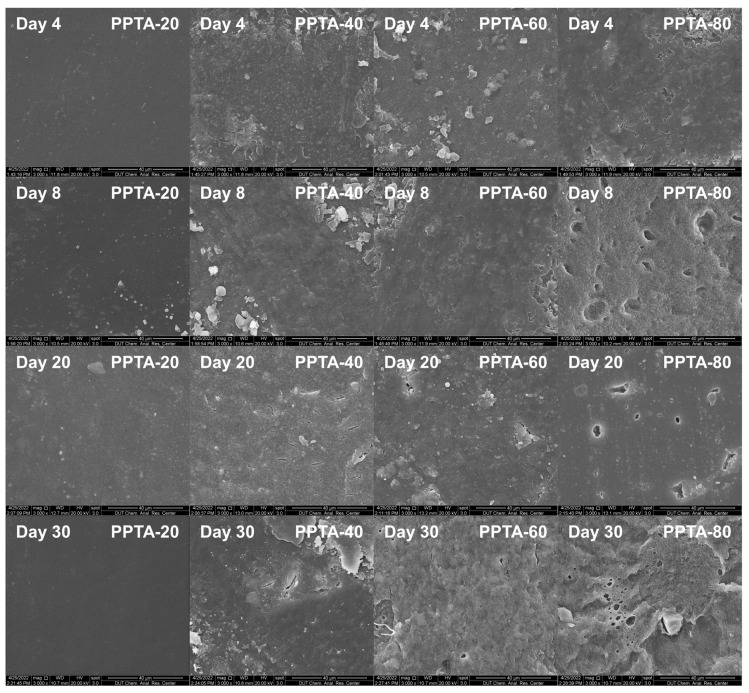
SEM diagrams of PPTA surface under different times of enzyme degradation.

The surface electron micrographs of the copolymers with different degradation cycles are shown in Figure 7. The surface of the PPTA-20 sample was smooth and did not show cracks or holes, indicating that no degradation behavior occurred. PPTA-40 showed more obvious cracks after 20 days of degradation, but its surface was flatter compared to PPTA-60 and PPTA-80, which was due to the fact that in semi-crystalline polymers, the enzyme degradation rate was higher because the insertion of PA blocks disrupted the regularity of the molecular chains and thus reduced the crystallization ability of the copolymer. This is because in semi-crystalline polymers, enzymatic degradation reactions preferentially occur in the amorphous region [35], whereas PPTA-60 and PPTA-80 are amorphous polymers and are therefore susceptible to erosion to form larger pores. The proportion of the amorphous zone increases, and the crystalline structure of the crystalline zone is perfect, which becomes the skeleton to support the amorphous zone during the degradation process, thus forming larger erosion pores.

The thermal properties as well as the microstructure of the PPTA copolymers with different degradation cycles were investigated by DSC and ^1^H NMR, respectively. The degradation time, mass loss rate, and thermal property data of the samples after different times of degradation are summarized in Table 3. The chemical composition and thermal properties of the copolymers did not change significantly for different degradation times, thus indicating that the degradation process is a surface degradation mechanism [36,37].

## 4. Conclusions

Poly(propylene terephthalate-co-adipate) (PPTA) random copolymers with different compositions were synthesized using adipic acid as the third monomer for the copolymer modification of PPT. The effects of different components on the polymer properties were investigated. Additionally, ^1^H NMR analysis was used to determine the microstructure and composition of the copolymers. The introduction of the comonomer decreased the *T*_g_, *T*_m_, and crystallinity of the copolymers. The thermal decomposition temperature, thermal degradation activation energy, carbon residue, and reaction level of the copolymers decreased as the PA content increased, with the thermal degradation activation energy ranging from 297.8 to 302.1 kJ/mol, indicating good thermal stability. PPTA copolymers also exhibit hydrophobic behavior when the PA content is between 45.4% and 79.8%. The water contact angle gradually increases as the proportion of PA in the polymer increases. Furthermore, the introduction of the third monomer improved the enzymatic degradation rate of the copolymers to a certain extent. PPTA showed significant degradation within 30 days when the PA content in the copolymers exceeded 45.36%, and the copolymers displayed a higher degradation rate as the PA content increased. These properties suggest that PPTA copolymers could be applied in the field of biodegradable polymer materials.

## Figures and Tables

**Table 1:** Reaction conditions of PPTA copolymers.

Samples	n(PTA):n(AA)	Esterification Temperature (°C)	Esterification Time (h)	Polycondensation Temperature (°C)	Polycondensation Time (h)
PPT	100:0	230	4	250	5
PPTA-20	80:20	230	4	250	5
PPTA-40	60:40	230	4	250	5
PPTA-60	40:60	230	4	240	5
PPTA-80	20:80	230	4	230	5
PPA	0:100	230	4	230	5

**Table 2:** Composition and molecular data of PPTA copolymers.

Samples	n(PTA):n(AA)	^a^ Composition (%)		[*η*] (dL/g)	^b^ *M_n_* (10^4^)	*M*_w_/*M*_n_	*L_PT_*	*L_PA_*	*R*
		PT	PA						
PPT	100:0	100	0	1.06	—	—	—	—	—
PPTA-20	80:20	77.24	23.76	0.58	—	—	4.57	1.26	1.01
PPTA-40	60:40	54.64	45.36	0.60	—	—	2.17	1.81	1.01
PPTA-60	40:60	42.50	57.50	0.81	2.9	1.6	1.63	2.65	0.99
PPTA-80	20:80	20.20	79.80	0.71	1.7	2.3	1.25	5.54	0.98
PPA	0:100	0	100	0.30	0.8	2.0	—	—	—

^a^ The composition of the polymers was characterized by NMR. ^b^ *M_n_* was measured by GPC with the mobile phase THF.

**Table 3:** The thermal performance parameters of PPTA during the process of enzymatic degradation.

Samples	Time (Days)	Mass Loss (%)	^a^ PA Content in Copolymer (%)	^b^ Δ*H_m_* (J/g)	*T_g_* (°C)	*T_m_* (°C)
PPTA-20	0	0	23.76	32.72	61.9	203.8
	16	0	24.68	31.65	62.1	202.1
	20	0	24.77	32.51	63.5	203.6
	30	0	25.18	32.08	64.8	202.8
PPTA-40	0	0	45.36	26.25	−4.6	152.4
	16	0.50 ± 0.10	44.86	27.86	−3.2	151.5
	20	0.78 ± 0.15	45.12	25.16	−3.8	152.4
	30	1.23 ± 0.11	44.64	28.15	−4.1	152.8
PPTA-60	0	0	57.50	0.14	−11.0	107.6
	16	4.64 ± 0.21	58.12	0.58	−11.8	106.8
	20	5.32 ± 0.19	60.23	1.17	−12.0	105.3
	30	6.70 ± 0.15	61.66	0.52	−12.0	106.9
PPTA-80	0	0	79.80	—	−22.4	—
	16	26.62 ± 0.19	81.12	—	−23.3	—
	20	36.53 ± 2.15	80.62	—	−22.9	—
	30	59.10 ± 1.10	82.73	—	−22.3	—

^a^ PA content was calculated by ^1^H NMR. ^b^ Thermal performance data were measured by DSC.

## Data Availability

Data is contained within the article or Supplementary Material.

## Supplementary Materials

The following supporting information can be downloaded at: https://www.mdpi.com/article/10.3390/polym16182588/s1, Figure S1: The 1H-NMR spectrum of PPTA-60; Figure S2: WXRD curves of PPTA copolymers; Table S1: Non-isothermal data of PPTA copolymers; Table S2: Crystallization kinetics of PPTA samples with different composition; Table S3: Thermal degradation kinetics for PPTA with different compositions.
