# Supplementary material for: Partially Bio-Based and Biodegradable Poly(Propylene Terephthalate-Co-Adipate) Copolymers: Synthesis, Thermal Properties, and Enzymatic Degradation Behavior
Source: Polymers (Basel). 2024 Sep 13;16(18):2588. doi: 10.3390/polym16182588 (PMC11435256; doi:10.3390/polym16182588)
Supplement: Supplementary file 1 [file polymers-16-02588-s001.zip › polymers-3182998-supplementary.pdf]

SUPPORTING INFORMATION

Partially bio-based and biodegradable poly(propylene terephthalate-co-adipate) copolymers: synthesis, thermal properties, and enzymatic degradation behavior

Ping Song [1], Mingjun Li [2], Haonan Wang [2], Yi Cheng [2] and Zhiyong Wei [2,*]

[1] School of Materials Science and Engineering, North University of China, Taiyuan 030051, China; songping@nuc.edu.cn

[2] Department of Polymer Science and Engineering, School of Chemical Engineering, Dalian University of Technology, Dalian 116024, China; mingjun_li0629@163.com (M.L.); 15541141525@163.com (H.W.); cy193777@mail.dlut.edu.cn (Y.C.)

* Correspondence: zywei@dlut.edu.cn

Contents

Figure S1. The 1H-NMR spectrum of PPTA-60. 2

Figure S2. WXRD curves of PPTA copolymers. 3

Table S1. Non-isothermal data of PPTA copolymers. 4

Table S2. Crystallization kinetics of PPTA samples with different composition. 5

Table S3. Thermal degradation kinetics for PPTA with different compositions. 6

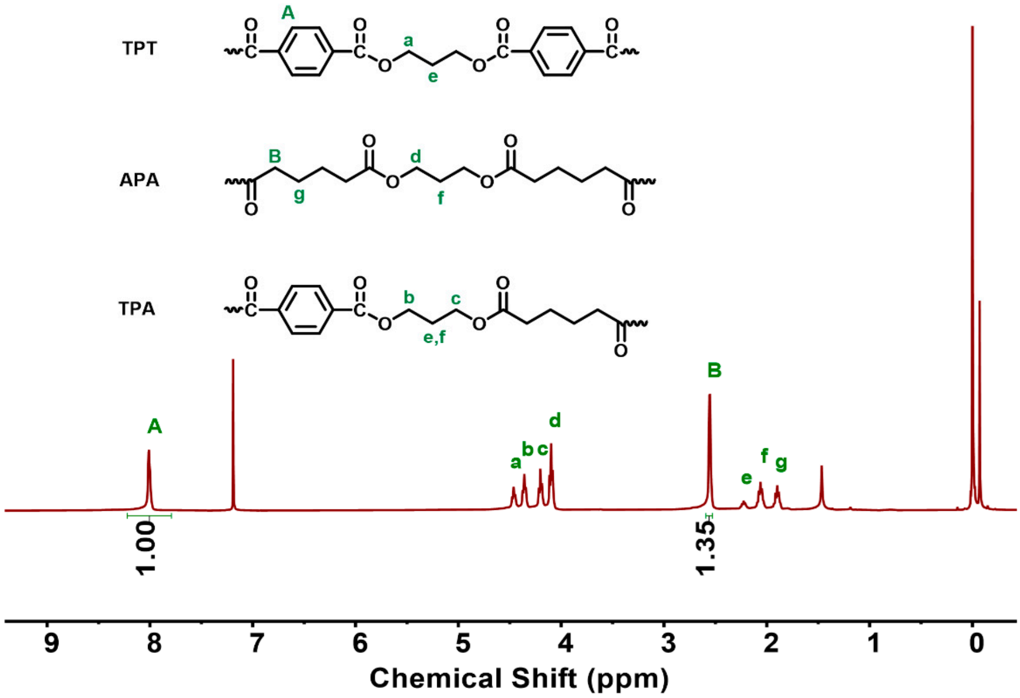

Figure S1. The 1H-NMR spectrum of PPTA-60.

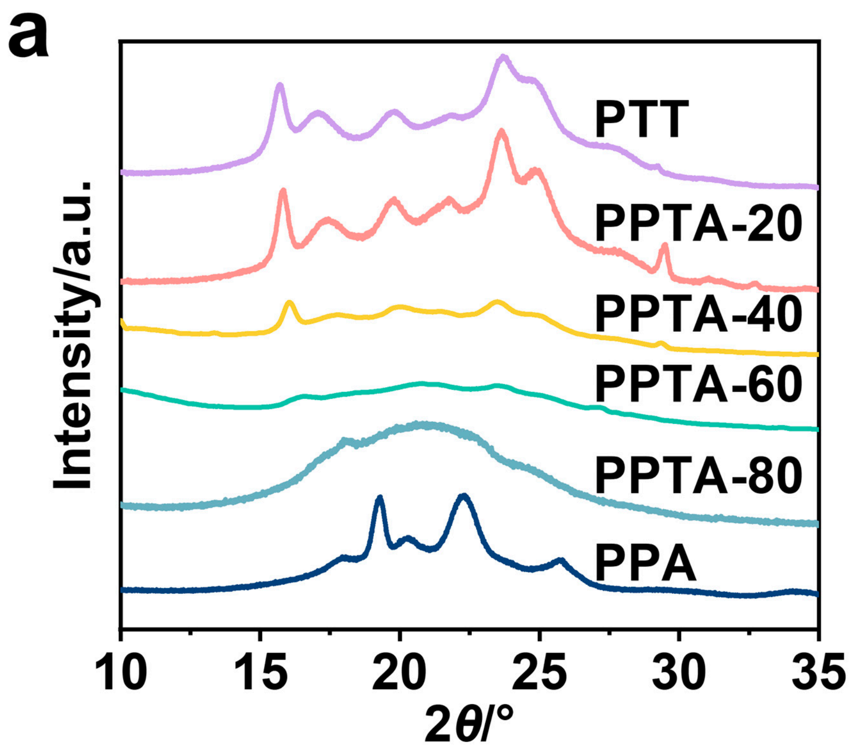

Figure S2. WXRD curves of PPTA copolymers.

Table S1. Non-isothermal data of PPTA copolymers.

Samples	T_m(°C)	T_c(°C)	T_g(°C)	$^aT_{cc}$(°C)	ΔH_m(J/g)	^bX_c(%)	^cX_c(%)
PPT	223.5	163.2	63.3	—	64.13	26.12	45.91
PPTA-20	203.8	148.9	61.9	—	32.27	18.16	23.10
PPTA-40	152.4	81.7	-4.6	—	26.25	13.59	18.79
PPTA-60	107.6	—	-11.0	-7.9	0.14	4.17	3.7
PPTA-80	—	—	-22.4	-20.1	0	0	0
PPA	100.7	—	-30.7	-29.0	0.44	6.83	0.31

(a) T_{cc} represents the temperature of the cold crystallization peak.

(b) Crystallinity was calculated by WAXD.

(c) Crystallinity was calculated by a DSC secondary heating curve.

Table S2. Crystallization kinetics of PPTA samples with different compositions.

Samples	T_c (°C)	n	k (min^{-n})	$t_{1/2}$ (min)	$\tau_{1/2}$ (min^{-1})	ΔE (KJ/mol)
PPTA-20	130	2.02	3.43	0.45	2.222	3.4
	132	2.02	2.20	0.56	1.786	
	134	2.03	1.45	0.69	1.449	
	136	2.01	1.01	0.83	1.205	
PPTA-40	130	2.07	0.16	2.27	0.441	159.1
	132	2.32	0.06	3.27	0.306	
	134	2.36	0.03	3.95	0.253	
	136	2.15	0.03	4.60	0.217	

Table S3. Thermal degradation kinetics for PPTA with different compositions.

Samples	T_d(°C)	T_{dm}(°C)	Carbon yield ratio(%)	n	ln$Z$(min^{-1})	E(KJ/mol)
PPT	370.9	403.1	9.32	1.35	56.1	302.1
PPTA-20	366.2	403.5	7.30	1.20	57.3	301.4
PPTA-40	364.1	406.7	4.38	1.03	55.5	299.3
PPTA-60	360.5	407.2	3.77	1.01	55.2	297.1
PPTA-80	357.7	408.1	2.00	1.00	55.1	297.6
PPA	351.8	408.6	0.97	0.93	62.0	297.8
